# The intersection of domestic abuse and menopause: a scoping review

**DOI:** 10.1186/s12905-025-04161-9

**Published:** 2025-11-27

**Authors:** C. Mann, K. Hinsliff-Smith, S. Olewe-Richards

**Affiliations:** 1 CHILL (Centre for Health Innovation, Leadership & Learning), NUBS University of Nottingham, Nottingham, UK; 2 De Montfort University, Leicester, UK; 3 Health & Life Sciences De Montfort University, Leicester, UK

**Keywords:** Domestic abuse, Domestic violence, Menopause, Women’s health, Review

## Abstract

**Background:**

The interplay between menopause and domestic abuse (DA) presents a complex, under-researched nexus within women’s health. Menopause, a significant physiological and psychosocial transition, may be affected by, or contribute to, women’s experiences of abuse. This scoping review explores how DA during the perimenopausal and postmenopausal stages, referred to as midlife (ages 40–65) impacts symptom severity, abuse dynamics, and healthcare engagement.

**Methods:**

A scoping review methodology following the JBI Manual for Evidence Synthesis and PRISMA-ScR guidelines. MEDLINE, CINAHL, Web of Science, and EMBASE were searched for English-language peer-reviewed studies exploring the relationship between menopause and DA with no restriction on date range. Inclusion criteria were studies involving women who were perimenopausal or postmenopausal and had experienced DA.

**Results:**

Of 189 unique records screened, 39 studies were included. Cross-sectional studies were most prevalent (*n* = 16, 41%), followed by cohort studies (*n* = 9, 23%) qualitative studies (*n* = 5, 15%) secondary data analysis (*n* = 3, 8%) and longitudinal studies (*n* = 2, 5%). There was also one each (*n* = 1, 2%) of clinical trial, retrospective analysis, case control study and systematic review.

Three interconnected themes were identified:(1) a consistent link between experiences of DA and increased severity of menopausal symptoms (*n* = 34); (2) a tendency for DA to escalate or (re)emerge during midlife and menopause (*n* = 5); and (3) missed opportunities for DA disclosure within menopause-related healthcare encounters (*n* = 14). Studies spanned 14 countries, with the majority conducted in the United States (*n* = 16). No studies from the UK were identified.

**Conclusion:**

For DA survivors there is an increase in menopausal symptoms, with profound effects on their mental, emotional, and physical health. Menopause represents both a potential risk period for DA and an opportunity for healthcare providers to identify abuse. This review highlights the urgent need for trauma-informed, menopause-sensitive healthcare practices, as well as further UK-based research.

**Supplementary Information:**

The online version contains supplementary material available at 10.1186/s12905-025-04161-9.

## Background

The menopausal transition represents a significant life stage for women, typically occurring between the ages of 45 and 55, and is characterised by hormonal fluctuations leading to the cessation of menstruation. This physiological process is often accompanied by a range of symptoms, including vasomotor disturbances (hot flushes and night sweats), sleep disruption, mood changes, and genitourinary symptoms. While these experiences vary considerably among women, they can significantly impact quality of life and wellbeing [4, 13]. This middle phase of adulthood represents a unique life stage distinct from both early adulthood and older adulthood, with its own developmental tasks and challenges [21]. Whilst the chronological boundaries are somewhat fluid, researchers generally recognise midlife as beginning around age 40 and extending to approximately age 65, though individual variations are substantial [24].

Domestic Abuse (DA) is defined as an incident or pattern of incidents of controlling, coercive, threatening, degrading and violent behaviour, including sexual violence, in the majority of cases by a partner or ex-partner and affects women across all life stages. Global estimates suggest that approximately one in three women will experience physical or sexual violence from an intimate partner during their lifetime [53]. Despite increasing recognition of DA as a public health concern, its specific impact during ageing and the menopausal transition has received comparatively little attention in research and clinical practice [29, 35].

The intersection of menopause and DA presents a complex terrain for investigation. Both experiences can involve significant physical, psychological, and social dimensions that may interact in ways that amplify negative outcomes for women. For example, the psychological distress associated with abuse may exacerbate menopausal symptoms, while physical and hormonal changes during menopause might influence how women experience and respond to abusive situations [1, 2]. Additionally, midlife, the age between 40 and 65, often brings other significant life transitions such as changes in family dynamics, caregiving responsibilities, and employment circumstances, potentially creating both additional vulnerabilities and opportunities for resilience [48]. The midlife stage refers to the developmental period typically spanning ages 40–65, characterised by distinctive biological, psychological, and social transitions [24]. For women specifically, this period encompasses significant reproductive changes, including perimenopause and menopause [40], coinciding with evolving family roles, career developments, and identity reassessment [35].

Healthcare settings represent crucial contexts where these intersecting experiences might be identified and addressed. However, research suggests that both menopause and DA remain under-recognised and inadequately addressed in many healthcare systems [20, 22]. Middle-aged and older women experiencing DA face particular barriers to disclosure and help-seeking, including age-related stigma, longer relationship histories, financial interdependence, and concerns about family disruption [29].

This scoping review aims to synthesise existing evidence on the relationship between DA and menopause, addressing a significant gap in the literature. By mapping the available evidence on symptom manifestation, lived experiences, and treatment approaches, this review seeks to inform more responsive healthcare practices and identify priorities for future research. Understanding this intersection is essential for developing appropriate interventions that address both the physiological aspects of menopause and the safety and well-being needs of women with lived abuse experience during this predicted life stage.

The review is particularly timely given increasing awareness of both menopause as a significant health concern requiring dedicated attention and DA as a persistent social problem affecting women's health across the lifespan. By bringing these two areas of concern together, we aim to contribute to a more nuanced understanding of women's midlife experiences and the development of more comprehensive approaches to care and support. Given variations in healthcare systems and support services across different countries and regions, we sought to map the geographical distribution of existing evidence. As UK-based researchers, we were particularly interested in understanding this phenomenon within a UK context.

## Methods

This scoping review was conducted in accordance with the JBI methodology for scoping reviews [38] and PRISMA-ScR [50]. This approach was chosen as the most relevant to the aim of understanding the niche field of combining DA with menopause and to meet the review objectives. The scoping review is suitable for exploratory work, to identify the evidence in this field, clarify key concepts in the literature, examine how research is conducted on this topic and identify gaps in the literature. A scoping review is an initial step for understanding a topic of interest and to identify future research opportunities [36].

Our analysis of the literature using Braun and Clark’s reflexive thematic approach [7] identified three distinct yet interconnected themes that illuminate different dimensions of women’s experiences when DA and menopause co-occur (1) the impact of abuse on symptom severity and presentation (2) the potential for abuse to escalate or emerge during the transition into menopause and (3) the opportunities and barriers present when identifying and supporting affected women within healthcare settings.

### Research questions

Based on our interest in exploring this area, we devised the following research questions: what are the experiences of women who have experienced DA during the (peri and full) menopause? What are the healthcare needs of women who have experienced DA during the (peri and full) menopause? How does the evidence help us to understand women survivors of DA during the (peri and full) menopause?

These research questions provided the framework for our comprehensive exploration of the literature.

### Inclusion criteria

#### Participants

Studies were eligible for inclusion if they involved women identified as experiencing or having experienced (in the past) a form of DA and also experiencing (presently) or having experienced (in the past) (peri or full) menopause before or during the study. If menopause studies included multiple patient groups, a clear subgroup analysis for women experiencing DA needed to exist. Otherwise, these studies were excluded. If studies related to DA included multiple patient groups, a clear subgroup analysis for women experiencing menopause needed to exist. Otherwise, these studies were excluded. No further age, socio-demographic variables, ethnicity or religion restrictions were made.

### Concept

Initial screening of titles identified papers that suggested relationships between DA/violence and menopause experiences. Abstract screening further refined the selection to include only papers with findings specifically related to DA survivors' experiences of menopause.

### Search strategy

The selection process followed PRISMA guidelines for scoping reviews with an open date for searching. Three researchers (CM, SOR, KHS) defined the search terms collaboratively and iteratively. A scoping review protocol was not registered.

Four electronic databases were systematically searched: MEDLINE, CINAHL, Web of Science, and EMBASE. The search strategy combined terms related to menopause and DA/violence. We conducted the initial search in two stages.

The first part of the search string consisted of the terms “peri-menopause” OR “menopause” to describe the condition, using a wildcard symbol to also include menopausal. For menopause-related terms, we used: “menopaus*” (or “menopause,” “menopausal,” “perimenopause,” “perimenopausal”). The second part consisted of terms related to DA, such as ‘DA’ or ‘domestic violence’. The keyword function was used sparingly because words such as “menopause” or “abuse” alone resulted in too many unrelated articles. The search combined BOTH with AND operator within the title or abstract of the paper, as either term alone generated results which were too broad to be relevant. We initially included the term ‘trauma’ but withdrew this after the first search revealed generalised results.

Through iterative review and familiarisation with literature at the end of the first search, we recognised the limitations of terminology used to describe DA as UK-centric terms. Our initial review identified further terms used in global literature, including “IPA” (intimate partner abuse), “IPV” (intimate partner violence), with a follow-up search using the same terms. Table 1 confirms the search terms used for each review of the chosen databases.

**Table 1:** Search terms used within the scoping review

Search	Search term 1	Operator	Search term 2	Notes
1	menopaus*	AND	“domestic abuse”	
1	menopaus*	AND	“domestic violence”	
1	~ ~ menopaus* ~	AND	~ ~ trauma ~ ~	10 k + results. Withdrawn search term
1	Peri-menopaus*	AND	“domestic abuse”	
1	Peri-menopaus*	AND	“domestic violence”	
2	menopaus*	AND	“IPA”	
2	menopaus*	AND	“partner abuse”	
2	menopaus*	AND	“IPV”	
2	menopaus*	AND	“partner violence”	
2	Peri-menopaus*	AND	“IPA”	
2	Peri-menopaus*	AND	“partner abuse”	
2	Peri-menopaus*	AND	“IPV”	
2	Peri-menopaus*	AND	“partner violence”	

The first search commenced in November 2024 and concluded in December 2024; the second search took place in January 2025 with an open date range to capture all relevant studies. One researcher (CM) conducted the initial searches, yielding 639 initial results and a further 1,124 results in the follow-up search. After removing duplicates, 189 sources remained for further review.

**Fig. 1:**
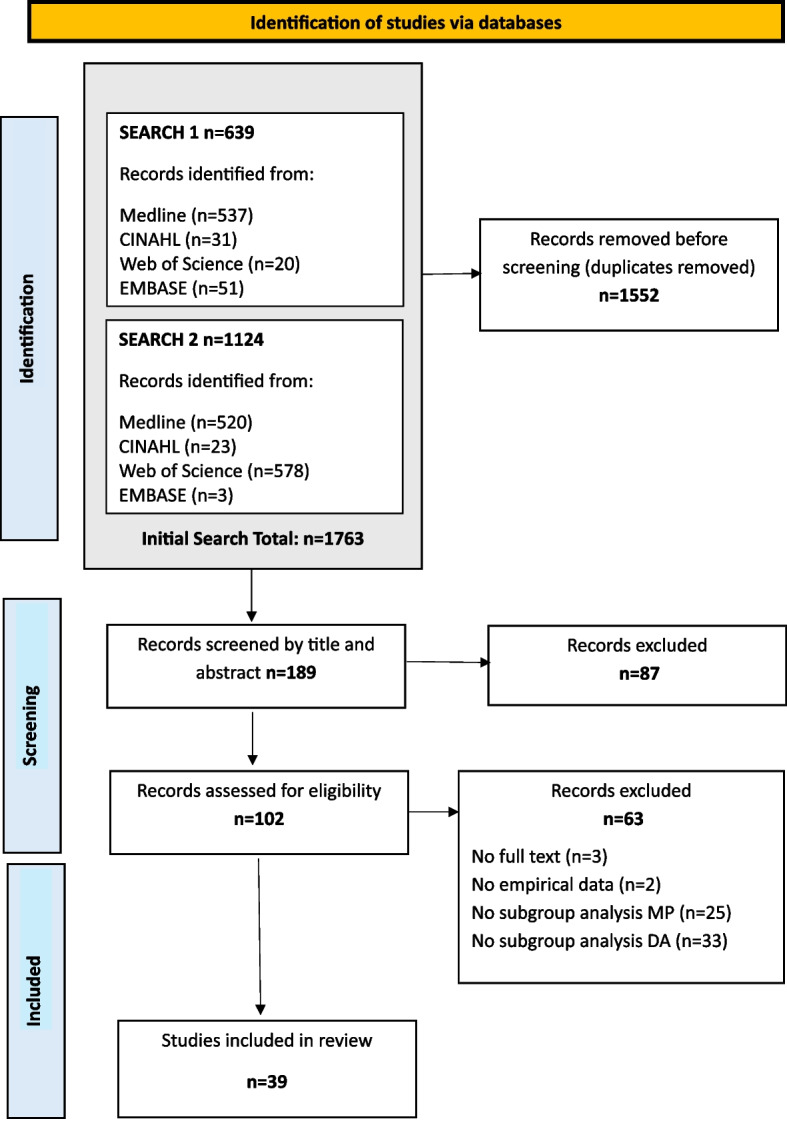
PRISMA diagram

Two researchers (CM, SOR) independently reviewed all abstracts and came to consensus resulting in 102 articles selected for eligibility review. Full-text assessment for eligibility was conducted by all three researchers (CM, SOR, KHS) each reviewing all articles and achieving consensus on eligibility, resulting in a final sample of 39 articles for data extraction and analysis. Figure 1 is a PRISMA diagram outlining the stages of the scoping review.

### Data extraction and analysis

A standardised data extraction form was developed to systematically record key information from each included study. Data extracted included: study design, participant characteristics, menopause-related findings, DA context, and key outcomes related to the intersection of menopause and DA experiences. Following standard scoping review methodology, quality assessment of the studies was not undertaken as we aimed to map existing evidence rather than evaluate methodological quality.

The extracted data was compiled in a spreadsheet and analysed thematically to identify patterns, gaps, and areas of consensus in the existing literature. We employed an inductive approach to the analysis of our literature data, with constant reviewing and reframing and agreeing upon.

## Results

In this section we will summarise the characteristics of included studies before exploring the three key thematic areas which were generated from exploring the content of the included papers. These themes are the impact of DA on menopause symptoms, escalation or emergence of DA at midlife and missed opportunities for DA disclosure in menopause care.

### Characteristics of included studies

Our review identified 39 studies examining the relationship between DA and menopause. These studies represented diverse geographical contexts, methodological approaches, and participant populations.

#### Geographical distribution

The included studies spanned 14 countries across multiple continents. The largest proportion originated from the United States of America (*n* = 17, 44%), followed by Australia (*n* = 4), Iran and Spain (*n* = 3), and Brazil and China (*n* = 2). Other countries represented included Chile, Ecuador, Ethiopia, Germany, India, Pakistan, Mexico, and Turkey with one study each. This geographical diversity enabled examination of the phenomenon across varied cultural and healthcare contexts.

#### Study designs

The methodological approaches varied considerably. Cross-sectional studies were most prevalent (*n* = 16, 41%), followed by cohort studies (*n* = 9, 23%) qualitative studies (*n* = 5, 15%) secondary data analysis (*n* = 3, 8%) and longitudinal studies (*n* = 2, 5%). There was also one each (*n* = 1, 2%) of clinical trial, retrospective analysis, case control study and systematic review. This methodological diversity provides both breadth and depth of understanding to this review.

#### Participant characteristics

Collectively, the studies included approximately 85,000 participants. Sample sizes ranged from 14 (qualitative interviews) to 34,282 (national health surveys, [8]). Most studies focused specifically on women in the menopausal transition or post-menopausal women, typically aged 40–65 years. Several studies utilised data from large national cohorts such as the Study of Women's Health Across the Nation (SWAN) in the United States [16] and national demographic health surveys in countries including Ethiopia [30] and Brazil [8].

**Fig. 2:**
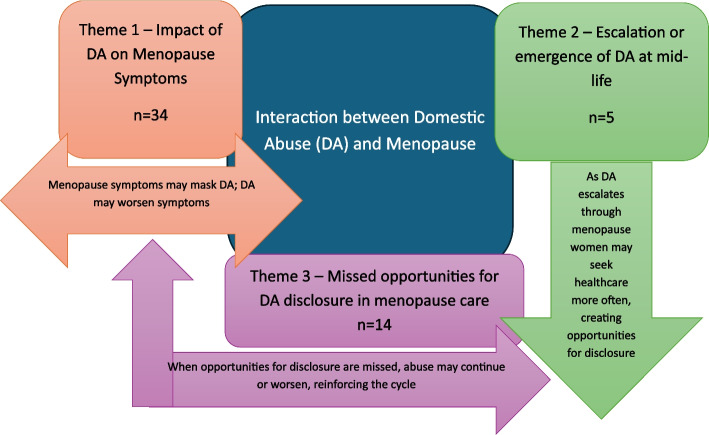
Thematic links in literature about menopause and domestic abuse

Braun and Clark’s (2006, 2017, 2022) six-step process was followed: ‘familiarizing yourself with your data’, ‘generating initial codes’, ‘searching for themes’ ‘reviewing themes’, ‘defining and naming themes’ and ‘producing the report’. We identified three primary themes in the literature: (1) Impact of DA on menopause symptom severity, (2) Escalation or emergence of DA at midlife, and (3) Missed opportunities for DA disclosure in menopause care. These themes represent distinct but interconnected dimensions of the relationship between DA and menopause. Figure 2 outlines the thematic links identified.

The table outlining the 39 sources and the themes against each paper is held in Supplementary Data SD1.

##### Theme 1: impact of DA on menopause symptom severity

 The largest category of studies (*n* = 34, 89%) focused on the association between DA and health outcomes related to menopausal symptoms. These studies consistently documented that woman with histories of DA experienced more severe menopausal symptoms across multiple domains, including vasomotor, psychological, and genitourinary symptoms.

The foundational work in this area began with studies examining the relationship between abuse and hormonal function. Allsworth et al. [1, 4] conducted pioneering research demonstrating that violence victimisation was associated with altered ovarian hormone levels and accelerated onset of perimenopause. Their 2001 study found that physical and sexual abuse may lead to neuroendocrine disruption affecting ovarian function, while their 2004 longitudinal analysis showed that violence, especially early in life, may affect the age of perimenopause via stress response dysregulation. These biological mechanisms help explain the consistent association between abuse and early menopause onset found in subsequent studies.

Several large-scale epidemiological studies identified specific symptom patterns. Gibson et al. [18] found that emotional intimate partner violence (IPV) was associated with increased odds of sleep disturbances (OR 1.36), night sweats (OR 1.50), and pain during intercourse (OR 1.60). In a different study, Gibson et al. [19] further demonstrated that interpersonal trauma had specific impacts on ageing-related genitourinary symptoms, with sexual assault being associated with vaginal symptoms and emotional abuse influencing multiple symptoms. Vegunta et al. [51] reported that women experiencing recent abuse had significantly higher menopausal symptom burdens, particularly for psychological symptoms, and suggested that the presence of severe menopausal symptoms, in the appropriate context, may prompt health care providers to ask about potential abuse and to offer support resources.

The relationship between DA and mental health symptoms during menopause was thoroughly documented across multiple studies. Chedraui et al. [10] found that depressive symptoms were highly prevalent among climacteric women and were strongly associated with menopausal symptoms and partner factors such as unfaithfulness and alcohol abuse. Arslantas et al. [3] found that domestic violence history was associated with more than twice the odds of depression (OR 2.168) in postmenopausal women. Carpena et al. [8] reported similar findings for both depression (OR 2.36) and suicidal ideation (OR 2.02), with their mediation analysis showing that 10.6% of the association between sex and major depressive episodes could be explained by violence exposure. Mitchell and Woods [34] found that a history of sexual abuse was significantly associated with depressed mood severity during the menopausal transition, highlighting the importance of considering the context of women’s lives when assessing mental health symptoms during this period.

Studies examining broader health impacts demonstrated concerning patterns. Mishra et al. [33] identified associations between IPV and earlier onset of natural menopause, with smoking as a mediating factor in this relationship. Rocca et al. [39] reported links between childhood or adult abuse and increased risk of bilateral oophorectomy before natural menopause, suggesting that abuse history might influence surgical decision-making. Thurston et al. [49] found that interpersonal violence exposure doubled the risk of cardiovascular disease events in midlife women, with systolic blood pressure being an important mechanism in this association. Woods et al. [52] documented that intimately abused women reported numerous physical health symptoms that could be misconstrued as normal menopausal symptoms, complicating differential diagnosis by healthcare professionals.

Studies focusing specifically on menopausal symptom severity consistently showed amplified effects among abuse survivors. Schei et al. [42] conducted an eleven-year prospective study of Australian women and found that IPV-exposed women had a higher number of bothersome symptoms, though they noted that vaso-motor symptoms specifically were not reported more often by IPV survivors. Sourinejad et al. [45] found that severity of menopausal symptoms increased with the severity of psychological, physical, and sexual violence experienced, with impacts extending to quality-of-life measures. Shariatmoghani and Nasiri [44] further supported this relationship in their cross-sectional study of 250 menopausal women in Iran, finding significant direct relationships between violence and mental, physical, and emotional dimensions of menopausal experiences. Their research particularly highlighted the impact of sexual dimensions of violence on menopausal experiences. Mendoza-Huertas et al. [32] identified that women affected by violence had more intense menopausal symptoms, an earlier age at menopause by approximately 20 months, and reported a worse quality of life during menopause compared to women who had not experienced violence. Faleschini et al. [14] reported that a history of physical abuse (reported by 37.3% of participants) was associated with worse menopausal symptoms in both somato-vegetative and psychological domains, along with worse general health and greater depressive symptoms.

The impact of DA on sexual function during menopause received specific attention in several studies. Martínez-Madrid et al. [28] found that the lowest sexual function scores (indicating worse function) were negatively associated with IPV, depression, and other female health issues. They identified IPV as a key factor contributing to deteriorating sexual health in middle-aged women. Teixeira de Araujo Moraes [47] similarly found that women who had suffered domestic or sexual violence displayed higher numbers of comorbidities, including unsatisfactory sexual life, during the climacteric period. Pérez-Roncero et al. [37] reported that menopausal symptoms in middle-aged Spanish women were related to menopausal status and partner factors, including gender violence.

Studies examining specific symptom clusters found nuanced relationships. Kling et al. [23] found that relationship distress was associated with more severe menopausal symptoms, particularly in the psychological domain. Conklin and Karakurt [11] reported that women who experienced more relationship conflict and violence had more frequent vasomotor symptoms and rated their hot flashes and night sweats as more severe. A cognitive behavioural therapy (CBT) intervention showed greater benefits for women with IPV backgrounds, particularly in developing negotiation skills. Garcia et al. [16] found that violence and traumatic stress against midlife women were associated with higher-than-normal changes in weight and waist circumference, both increases and decreases, as well as symptoms of anxiety, depression, and cognitive problems.

Studies from diverse global contexts confirmed the consistent relationship between abuse and symptom severity. Schwarz et al. [43] conducted a population-based survey in Germany, finding that experience of abuse was associated with both prevalence and intensity of menopausal symptoms. Barzin et al. [5] found that 32.1% of middle-aged Iranian women experienced violence, with psychological violence being most common (30.5%), and identified a significant association with depression. del Carmen Macías-Cortés et al. [12] conducted a randomised, placebo-controlled trial in Mexico with 133 climacteric women experiencing moderate-to-severe depression, examining the association between domestic violence, sexual abuse, and marital dissatisfaction and response to depression treatment. Sattar [41] interviewed women in a Pakistani shelter home and found that 17 of 21 participants reported restrictive fertility and early menopause as major reproductive health problems related to IPV. Fu et al. [15] analysed Chinese national data showing that women aged 50–54 experienced a greater burden of IPV compared to other age groups, linking this to emotional instability due to menopause and coinciding with typical retirement age for Chinese women.

Overall, these studies present wide-ranging evidence that DA has broad and profound effects on women’s experiences of menopause, from biological impacts on the timing of menopause to psychological distress, physical symptom severity, and overall quality of life during this transition.

##### Theme 2: escalation or emergence of DA at midlife

Five studies (13%) specifically documented the escalation or emergence of DA during the midlife and menopausal period. These studies provided insights into how relationship dynamics might shift during this life transition, potentially increasing vulnerability to abuse.

Strezova et al. [46] conducted a qualitative study with 81 Macedonian women in Australia, finding a recurring theme of men’s attitudes toward their wives changing after menopause occurred. The women reported that their husbands sometimes began treating them as "non-sexual" after menopause, which women experienced as a form of rejection rather than liberation. This shift in male attitudes was described as a precipitating factor in DV, extramarital affairs, and divorce. The study highlighted those cultural expectations about women’s roles and value could interact with menopause to create particular vulnerabilities to domestic abuse and IPV.

Li et al. [25] documented how Chinese menopausal women experienced increased physical and verbal abuse within their families after menopause through in-depth interviews with fourteen menopausal women aged 48 to 53 years. One participant explicitly stated: "After I went through menopause, my husband became significantly worse towards me." The researchers linked this to cultural stigma surrounding menopause and patriarchal family structures. The phenomenological qualitative research design revealed that women faced stigmatisation within families primarily due to traditional gender norms and a lack of understanding about menopausal symptoms, creating an environment where abuse could escalate.

Mekonnen and Balemual [30] analysed data from the 2016 Ethiopia Demographic and Health Survey. The work found that 31.8% of midlife women (aged 35–49) reported experiencing spousal violence. Their analysis of 1,628 midlife women concluded that spousal violence amongst these women represented a global public health concern, with similar prevalence rates reported across different countries and cultures [30]. This research identified specific risk factors for midlife women for potential spousal violence combined with rural residence, lower educational status, and partners’ alcohol consumption. This study provides insights into contextual factors that might influence abuse during midlife and around the time of peri-menopause.

Thomas et al. [48] examined responses from 81 midlife women in the Seattle midlife Women’s Health Study who were asked about the most challenging aspects of their lives. While few women explicitly mentioned menopause as their primary challenge, many reported experiencing multiple concurrent stressors, including relationship problems, at midlife. The study found that women experienced significant challenges related to "relationship with partner" during this life stage, including instances of abuse that intensified with midlife transitions. The researchers noted that women found themselves searching for balance amid multiple co-occurring stressors while coping with losses and transitions, sometimes in a context of violence.

Mendez et al. [31] conducted a qualitative study with 11 Mexican women in Albuquerque, New Mexico, documenting their experiences with domestic violence. While the study primarily focused on a community-driven approach to supporting Latinas with various mental and behavioural health issues, it captured important insights about abuse during menopause. One participant directly linked menopause to an escalation in abuse.

This collection of evidence suggests that the menopausal transition may represent a period of heightened risk for some women, potentially related to changing relationship dynamics, cultural attitudes toward ageing women, and the stress of multiple concurrent life transitions. The research highlights the importance of considering the sociocultural context of menopause when assessing abuse risk, as patriarchal attitudes and stigma surrounding this life transition may contribute to increased vulnerability in some settings. While limited in number, these studies provide clear evidence that abuse may occur or intensify during the menopausal transition and identify this as an important area for further research and intervention.

##### Theme 3: missed opportunities for DA disclosure in menopause care

Fourteen studies (36%) highlighted missed opportunities for identifying DA in healthcare settings during menopause care. These studies documented both women's reluctance to disclose abuse when receiving menopause-related healthcare and healthcare providers’ lack of attention to potential abuse.

Loxton et al. [26] analysed data from 14,100 women aged 45–50 years in the Australian Longitudinal Study on Women’s Health found that women who had experienced DV sought general practitioner consultations more frequently than women without such experiences, suggesting they may be seeking consultations for reasons beyond their apparent health status. Despite this increased contact with healthcare services, DA often remained undisclosed. The researchers noted that physical and psychological health status only partially explained the increased health service use associated with DV, indicating additional motivations for seeking care.

Schei et al. [42] conducted an eleven-year prospective study with 438 Australian-born women aged 45–55 years and identified IPV as a significant contributing factor in the development of ill health. The researchers emphasised that clinicians should be aware of IPV’s contribution to health outcomes and investigate whether it is present when assessing clients. This highlights a critical opportunity for healthcare providers to identify and address abuse during routine menopause care.

Woods et al. [52] studied 157 currently abused women and noted that many common physical health symptoms reported by abused women are similar to normal ageing and menopausal processes, making it challenging for practitioners to distinguish between symptoms related to menopause and those related to abuse without specific screening. They emphasised that assessment and intervention are complicated by time constraints in the current healthcare delivery system and recommended more comprehensive health histories to capture symptom patterns over time.

Cerulli et al. [9] conducted a cross-sectional secondary analysis of a 2006 USA telephone survey with 1,881 subjects and pointed out that the increasing provision of menopause education in healthcare settings creates opportunities for private patient-to-healthcare provider contact where patients might report IPV. The researchers specifically mentioned pharmacies as potential portals for public health promotion, highlighting menopause education services as possible contexts for IPV disclosure, but questioned whether professionals are prepared for these disclosures.

Teixeira de Araujo Moraes et al. [47] studied 124 women aged 40 to 65 years who had experienced domestic and/or sexual violence, comparing them with a control group of 124 women who had not. They highlighted that 80.6% of women experiencing violence during the climacteric period did not seek healthcare services despite having higher numbers of co-morbidities and more severe menopausal symptoms than women not exposed to violence. They argued that health services could become "privileged spaces where professionals can break the covenant of silence and the cycle of violence" (p.257) if properly trained, emphasising that "the interference of the health professional at ‘windows of opportunity’ are essential for the rupture of the process of violence"(p.256).

Gibson et al. [18] studied 2,016 women aged 40 to 80 years and emphasised the need for greater recognition of traumatic exposures by clinicians caring for midlife and older women. They found that lifetime history of IPV or sexual assault and current clinically significant symptoms of PTSD are common and associated with menopause symptoms. The researchers recommended routine screening for traumatic exposures and trauma-informed care approaches for symptoms related to menopause and ageing. In a related study, Gibson et al. [19] analysed data from 1,551 older women and found that emotional abuse was common but often under-recognised in clinical settings, despite having independent effects on genitourinary symptoms that may negatively impact quality of life.

Makaroun et al. [27] examined data from 4,481 women aged 45 and older including 2,937 aged 45–59, and found that middle-aged women screening positive for IPV had more than twice the odds of having a diagnosis of depression, anxiety, PTSD, or substance use disorder, and nearly 4 times the odds of suicidal behaviours or self-harm. The researchers emphasised that visits to doctors present important opportunities to screen for DA concerns in healthcare settings and that failing to assess for IPV may result in missing a key contributor to mental health conditions.

Mitchell and Woods [34] studied 291 women from the Seattle midlife Women’s Health Study and found that factors reflecting the context of women's lives, including a history of sexual abuse, were associated with the severity of depressed mood during the menopausal transition. They emphasised that clinicians need to consider the context in which midlife women experience the menopausal transition and mood symptoms beyond just hormonal changes, highlighting an opportunity to address abuse history during menopause care.

Binfa et al. [6] interviewed 22 midwives working in primary health care clinics and 13 midwifery students with clinical experience in Chile. The midwives reported that women in midlife had disclosed experiences of absent or aggressive partnerships during healthcare visits. The midwives recognised that they lacked competence in addressing the psychological and social healthcare needs of women in midlife related to violence, abuse, and sexuality issues, despite considering themselves the most appropriate health staff to provide care for women in midlife.

Barzin et al. [5] surveyed 1,266 middle-aged women (45–64 years) randomly selected from six health centres in Ahvaz, Iran, and found that 32.1% experienced violence, with rates increasing with age, especially for women aged 45 to 49. The researchers concluded that a major factor in depression among these women was aggressive behaviour by their husbands, suggesting that healthcare providers should be alert to the possibility of abuse when middle-aged women present with depression.

Vegunta et al. [51] analysed data from 3,740 women and found that those reporting abuse (physical, emotional or sexual) in the last year had significantly higher mean total menopausal symptom bother scores. The researchers suggested that this association could serve as a prompt for providers to ask about current abuse when women present with severe menopausal symptoms, representing an opportunity for identification and intervention.

Carpena et al. [8] analysed data from a national health survey including 34,282 women (17.5% aged 45–54), reporting that violence explained 10.6% of the association between sex and major depressive episodes and 8.0% of the association between sex and suicidal ideation. The researchers highlighted the importance of considering domestic violence when planning public mental health policies and clinical approaches for women, particularly during hormonal transitions like menopause.

George [17] conducted a qualitative ethnographic study of 190 women in a coastal fishing village in southwestern India found that while the women experienced physical and verbal abuse from their husbands (who often became violent when drunk), they did not associate symptoms they experienced, like depression, insomnia, and cold sweats, with menopause itself but rather with their life circumstances.

This evidence demonstrates a gap in healthcare provision, suggesting that women experiencing DA during menopause may be engaging with healthcare services without receiving appropriate support for abuse-related issues, and healthcare providers may lack the training and tools to identify and respond to DA in this population. The evidence points to multiple missed opportunities for intervention across diverse healthcare settings, from primary care to specialist menopause services, pharmacies, and midwifery care, highlighting the need for improved screening practices, provider training, and trauma-informed approaches to menopause care.

## Discussion

This scoping review examined the relationship between DA and menopause across 39 studies from diverse global contexts. The findings reveal complex interconnections between abuse exposure and menopausal experiences, with implications for understanding symptoms, lived experiences and treatment approaches.

The relationship between DA and menopausal symptoms requires careful interpretation regarding causation versus association. Whilst DA is associated with emotional and physical symptoms that reduce quality of life across the reproductive lifecycle, this review identified specific patterns during midlife that warrant distinct attention. The variety of study designs included in this review, from cross-sectional surveys to longitudinal cohort, provides different levels of evidence regarding these relationships. Cross-sectional studies can demonstrate associations but cannot establish causality, whilst longitudinal studies help clarify how these experiences unfold and interact over time. The emotional dimensions of menopausal experiences documented in our findings may reflect both the physiological changes of menopause and the psychological impact of abuse, creating complex symptom presentations that require nuanced clinical assessment to distinguish between symptoms attributable to abuse versus those related to the natural menopausal transition.

This scoping review aimed to explore the experiences and needs of women during menopause who have experienced DA. The findings indicate that evidence predominantly addresses these as individual issues, with less attention given to this population as a unique group.

Our analysis, guided by the three research questions exploring women’s experiences and healthcare needs, revealed interconnected themes that highlight the complex relationship between DA and menopause. The findings demonstrate that women navigating both DA and menopause experience amplified symptoms, may face escalating abuse during the menopause transition and encounter significant barriers to disclosing and seeking the right support within healthcare settings. Together, these themes underscore that woman experiencing both DA and menopause have distinct needs that are inadequately addressed when these issues are treated separately.

The studies included do give a unique and early insight into the needs and behaviours of women who have the multiple challenges of DA and the menopause. Although countries are globally represented, the majority of studies are in the USA, and a gap exists in Europe, particularly in the UK, relating to the needs and experiences of this population. This absence of studies from the UK is particularly significant given the UK’s distinct healthcare system and support services structure. UK policy frameworks around both menopause and DA (Domestic Abuse Act, 2021) create a unique context that may influence women’s experiences of help-seeking behaviour. The NHS provides universal healthcare, including specialist menopause services, whilst a network of statutory and voluntary sector organisations delivers DA support. To better understand how these services could identify and support women experiencing both DA and menopause, there is a need for UK-specific evidence. This gap in UK-based research limits our ability to develop evidence-based recommendations for practice within the UK healthcare and support systems. Overall, it is clear that more research is needed into women’s experiences of the menopause, with a specific focus on those who have also experienced or are presently experiencing DA. Adopting a review that was taken through a feminist lens to explore, understand and contribute to the wider sphere of health inequalities may be a useful juncture for further UK-based studies, which appear very poorly represented.

The results show evidence of consistent associations between DA and increased severity of all menopausal symptoms but with mental health impacts particularly pronounced. Several studies [1, 2, 33] identified a potential biological pathway whereby chronic stress from DA exposure may influence hormonal regulation and accelerate reproductive ageing. The experiential dimension of menopause for women exposed to DA revealed many similarities in experiences globally but also some distinct cultural and social patterns. One consistency across studies at the intersection of DA and help-seeking behaviours was that women in this population were less likely to seek help [26, 47]. This clearly identifies a gap for further work to explore, understand and change health-seeking behaviours in this population with potentially greater need. A small number of studies considering the efficacy of psychological or holistic therapies proved successful for this population, offering interesting areas for further research [12, 44].

The results of this scoping review show that women going through menopause who have also survived DA may experience early, widespread and highly impacting physical and mental health symptoms during their menopause journey.

### Further research

Future research needs to seek further understanding of how abuse exposure influences the menopausal transition over time, potentially through large-scale longitudinal studies, such as the SWAN study in the USA [16]. There is a clear need for the development of research of any type focusing on this population in the UK. Further research is needed to address the dual challenges of trauma recovery and symptom management, such as intervention studies specifically targeting this population. Studies that explore how healthcare systems can better identify and support menopausal women experiencing DA needs urgent examination, particularly given the documented need and reluctance of women to seek help as shown in this review. Additionally, research examining whether the relationship between DA and symptom severity differs between perimenopausal and postmenopausal women would provide valuable insights into how these experiences vary across the menopausal transition.

### Implications for policy and practice

The findings of this review have important implications for healthcare providers, researchers, and policymakers. For clinicians, they underscore the importance of integrating DA awareness into midlife women's healthcare and considering how abuse exposure might influence symptom presentation and management. For researchers the review highlights the need for further work in the UK and for robust methodological approaches, including longitudinal designs and intervention studies that address the unique needs of menopausal women experiencing DA. For policymakers, this review emphasises the importance of developing integrated approaches to women's midlife health that acknowledges the interconnected nature of physiological, psychological, and social dimensions of wellbeing.

Future directions should include the development and evaluation of trauma-informed approaches to menopausal healthcare, exploration of how intersecting identities and social positions influence these experiences, and greater attention to women's own perspectives regarding their needs and priorities during this life stage. Additionally, the review highlights the need to address the documented reluctance of women experiencing abuse to seek help during menopause, potentially through improved screening, enhanced provider training, and more accessible support services.

These findings highlight the importance of integrating DA screening into midlife women's healthcare and developing trauma-informed approaches to menopausal care. Healthcare providers should recognise that symptom presentations may be influenced by abuse exposure and adjust assessment and treatment approaches accordingly.

### Strengths and limitations

This is the first scoping review to explore the needs and experience of women who have suffered DA as well as the stages of the menopause. This review draws on broad global data from a range of methodologies covering a large number of participants, which is important and recognises the magnitude of the concerns. This scoping review highlights the need for further evidence related to this population group, who have clearly identifiable and treatable health needs.

The work does have several limitations. At the macro level of the scoping review, it cannot be guaranteed that all relevant studies have been identified. The terms "abuse" and "trauma" were trialled but can be used in a variety of contexts and were not considered suitable to be a MeSH Term. We recognised the initial limitation of our first search terms, which we expanded through our second search, whilst recognising that more terms may remain that we did not explore. The screening process involved at least two reviewers; however, initial searches and refinement were carried out by a single reviewer, and as such, carries the risk of bias or error. Many studies did not specifically focus on the intersection of abuse and menopause but included relevant data within broader investigations. Additionally, most studies employed cross-sectional designs, limiting causal inferences about the relationship between abuse exposure and menopausal outcomes. Finally, we acknowledge that it is possible that past and current exposure to DA could have different impacts on menopause symptom severity and health seeking and this is an area that may require further investigation.

## Conclusion

This review contributes to a growing understanding of menopause not merely as a biological event but as a complex biopsychosocial transition that occurs within specific relational and social contexts. Women experiencing both DA and menopause face the double burden of amplified symptoms and often missed opportunities for support. The evidence calls for urgent action to enable healthcare providers to access training to recognise abuse during menopause care, whilst policymakers must develop integrated approaches rather than treating these issues in isolation. The absence of UK research is striking, and addressing this gap to enable the transformation of healthcare practice is essential to ensure that midlife does not remain a period of hidden suffering for abuse survivors.

## Supplementary Information

Supplementary Material 1.

## Data Availability

All data generated or analysed during this study are included in this published article [and its supplementary information files].
